# Integration of epigenetic regulatory mechanisms in heart failure

**DOI:** 10.1007/s00395-023-00986-3

**Published:** 2023-05-04

**Authors:** Miron Sopic, Emma L. Robinson, Costanza Emanueli, Prashant Srivastava, Claudio Angione, Carlo Gaetano, Gianluigi Condorelli, Fabio Martelli, Thierry Pedrazzini, Yvan Devaux, EU-CardioRNA COST Action CA17129 and EU-AtheroNET COST Action CA21153

**Affiliations:** 1 Department of Medical Biochemistry, Faculty of Pharmacy, University of Belgrade, Belgrade, Serbia; 2 Division of Cardiology, Department of Medicine, University of Colorado Anschutz Medical Campus, Aurora, CO 80045 USA; 3 National Heart & Lung Institute, Imperial College London, London, UK; 4 School of Computing, Engineering & Digital Technologies, Teesside University, Tees Valley, Middlesbrough, TS1 3BA UK; 5 Centre for Digital Innovation, Teesside University, Campus Heart, Tees Valley, Middlesbrough, TS1 3BX UK; 6 National Horizons Centre, Darlington, DL1 1HG UK; 7 Laboratorio di Epigenetica, Istituti Clinici Scientifici Maugeri IRCCS, Via Maugeri 10, 27100 Pavia, Italy; 8 IRCCS-Humanitas Research Hospital, Via Manzoni 56, 20089 Rozzano, MI Italy; 9 Institute of Genetic and Biomedical Research, National Research Council of Italy, Arnold-Heller-Str.3, 24105 Milan, Italy; 10 Molecular Cardiology Laboratory, IRCCS-Policlinico San Donato, Via Morandi 30, San Donato Milanese, 20097 Milan, Italy; 11 Experimental Cardiology Unit, Division of Cardiology, Department of Cardiovascular Medicine, University of Lausanne Medical School, 1011 Lausanne, Switzerland; 12 Cardiovascular Research Unit, Department of Population Health, Luxembourg Institute of Health, L-1445 Strassen, Luxembourg

**Keywords:** Epigenetics, Epitranscriptomics, Machine learning, Data integration

## Abstract

The number of “omics” approaches is continuously growing. Among others, epigenetics has appeared as an attractive area of investigation by the cardiovascular research community, notably considering its association with disease development. Complex diseases such as cardiovascular diseases have to be tackled using methods integrating different omics levels, so called “multi-omics” approaches. These approaches combine and co-analyze different levels of disease regulation. In this review, we present and discuss the role of epigenetic mechanisms in regulating gene expression and provide an integrated view of how these mechanisms are interlinked and regulate the development of cardiac disease, with a particular attention to heart failure. We focus on DNA, histone, and RNA modifications, and discuss the current methods and tools used for data integration and analysis. Enhancing the knowledge of these regulatory mechanisms may lead to novel therapeutic approaches and biomarkers for precision healthcare and improved clinical outcomes.

## Introduction

Despite the progress in healthcare in the past half of the century, according to the World Health Organization (WHO), cardiovascular disease (CVD) is still the number one cause of death worldwide. Heart failure is a main cardiovascular condition, both in terms of prevalence and in terms of impact to the population. It has been estimated that in developing countries the prevalence of diagnosed heart failure in the general population is 1–2%, meaning that 64.3 million people globally are currently living with heart failure [12, 41, 97]. Mortality rates in European countries range from about 7–16% in patients with a chronic form of the disease, and 22–37% in patients with acute disease [99]. The socioeconomic burden of heart failure is expected to worsen considerably due to an aging population worldwide. Depending on the form of cardiac stressor and phenotype of pathology, disease-associated remodelling of the heart can include hypertrophy of cardiomyocytes, fibroblast activation, proliferation and transformation into myofibroblasts, endothelial cell dysfunction and immune cell infiltration. All these elements of cellular remodelling can contribute to morphological and functional changes in the heart including interstitial and perivascular fibrosis, cardiomyocyte death, loss of elasticity, cardiac dilatation or stiffening and overall reduced cardiac output. Considering that heart failure encompass complex malfunctions of the heart and blood vessels a deeper understanding of the underlying molecular causes of pathological remodelling of the heart and blood vessels remains imperative in order to develop effective preventive medicine and treatment.

Epigenetics is an exciting field of research that offers new insights into the underlying mechanisms of disease. In particular, epigenetics is essential in understanding cardiac diseases. At its core, epigenetics refers to the heritable changes in gene expression that occur without altering the underlying DNA sequence. The term comes from the Greek words "epi" (above) and "genetics" (birth). This field of research has revealed that lifestyle, environmental and biological factors can influence gene expression, leading to altered cellular behaviour that can have profound health implications [51, 112]. However, the real importance of epigenetic mechanisms is related to the programming of cellular development and the specific identity of the different cell types.

As said, heart failure is a complex disorder, and epigenetics might provide a useful framework for understanding its pathogenesis. Numerous studies have identified epigenetic biomarkers of CVD risk, including changes in DNA methylation and histone modifications [15, 36, 126]. These epigenetic changes are associated with altered gene expression, which may contribute to the development of cardiac disfunction. For example, epigenetic modifications can regulate the expression of genes that are involved in inflammation and atherosclerosis, two processes that are known to aggravate cardiac function [20].

Epigenetics also plays a role in the expression of environmental and lifestyle factors that are associated with heart failure. For example, smoking, diet and physical inactivity can all cause epigenetic changes that are associated with the risk of heart failure development [52].

Risk factors contribute to epigenetic and gene expression changes in cells of the cardiovascular system that culminate in disease, in particular over long periods of time if pathological stressors persist. Gene regulation is not a linear on/off switch, but a dynamic, highly complex, multi-layer process that is responsive to signalling and metabolic cues. Epigenetics is the term used to encompass dynamic, reversible non-genetic mechanisms that regulate chromatin structure and function and the resulting transcriptome. Thus, in order to unravel the complex interplay between epigenetic regulators and to get a whole picture of gene regulation alterations in disease, it is essential to use highly integrative and interconnected network analyses. Current technological revolution and advancement of molecular biology and next-generation sequencing techniques enable us to identify the location and abundance of epigenetic variation such as DNA and histone modifications throughout the mammalian genome, DNA accessibility, higher-order chromatin structure, as well as the resulting transcriptome and modifications thereof. In order to put epigenetic mechanisms of regulation in a wider cardiovascular physiological context, it is now possible to integrate epigenetic datasets with other biological big data such as proteomics and metabolomics, thus creating multi-layer omics profiles. More recent advances in single-cell sequencing technologies have further widened our understanding of molecular and cellular remodelling in disease, as well as brought a number of new challenges along with it.

For instance, the integration of this overwhelming production of data is extremely difficult to achieve without computational tools. Several approaches for multi-omics data integration have been developed, highlighting the advantages of integrating different levels of data with bioinformatics and machine learning approaches. Furthermore, the latest developments in machine learning and artificial intelligence have enabled significant progress in the field. It is now possible to explore inter-relationships between different layers of biological complexity through the construction of multi-modal machine learning systems, also taking into account mechanistically derived biological features [1, 78, 102]. This new approach provides a powerful tool for the investigation of novel players in cardiovascular pathology and can lead to the discovery of novel druggable targets and/or biomarkers. At the single-cell level, it is equally evident that an integrative analysis of several omics data will enable a more comprehensive view of cardiovascular disease molecular features compared to using only one omic layer. This has fuelled the recent development of single-cell multi-omic technologies and related computational tools [65].

In this review, we address how DNA, histone, and RNA modifications influence cardiac physiology and disease with a particular focus on heart failure. We discuss the current methods and tools used to analyse and integrate the gleaned information. Since epigenetics in vascular disease has been covered in recent reviews [106, 131, 139], this article will not review and discuss the role of epigenetics mechanisms in the vascular bed. Also, the relevance of ncRNAs and RNA-based epigenetic mechanisms in cardiac diseases has been discussed in details elsewhere [94].

## Overview of mechanisms of gene regulation

**Fig. 1:**
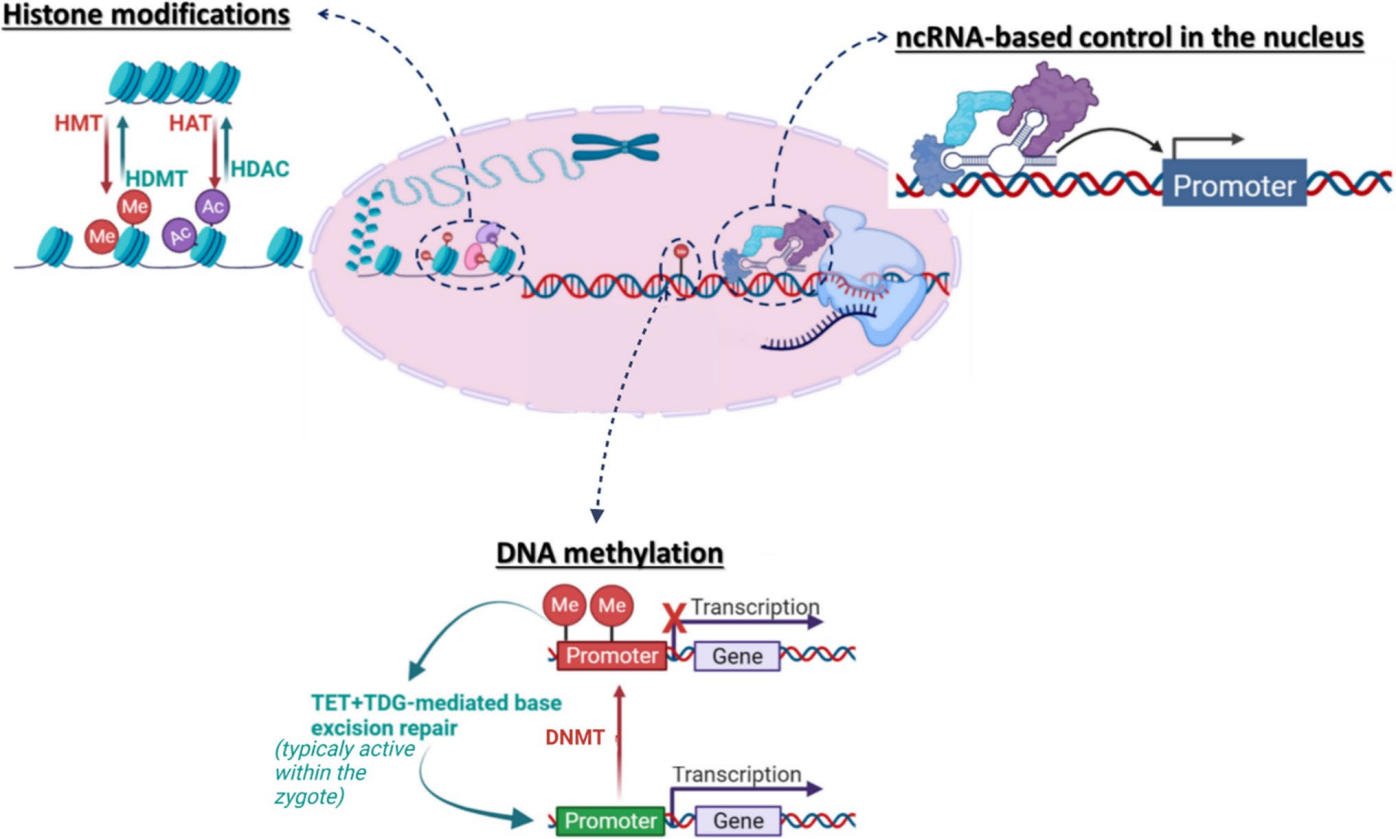
Key players in DNA-based epigenetic mechanisms of gene regulation including DNA methylation, histone modifications and ncRNA-based regulation (created by Biorender). *HMT* histone methyltransferase,* HAT* histone acetyltransferase, *HDMT* histone demethylase, *HDAC* histone deacetylase,* DNMT* DNA methyltransferases,* TET* ten-eleven translocation methylcytosine dioxygenases, TDG thymine DNA glycosylase

Many factors regulate gene activity at several levels in both the nucleus and the cytoplasm. Epigenomics is a recently developing field of research aimed at defining rules of gene expression regulation/function through the action of a plethora of specific enzymes and binding proteins named *erasers*, *readers* and *writers* [32]. Along with guidance from long non-coding RNAs (lncRNAs), the effect results in hundreds of post-translational modifications to DNA or histone tails, affecting wholesale chromatin conformation at the nucleosomal level, affecting the accessibility of genomic elements to RNA polymerase and other transcription factors. The major epigenetic regulatory mechanisms discussed below include DNA methylation and histone modifications (Fig. 1). RNA-based epigenetic control represents an additional layer of gene regulation at the level of transcription or post-transcriptionally. This review will not address the roles of ncRNAs in heart failure, as it has been extensively covered elsewhere [35].

DNA methylation refers to the addition of a methyl group to 5′ position of cytosine residues (5mC) throughout the genome. DNA methylation is *written* by the family of enzymes known as DNA methyltransferases (DNMT1, DNMT2, and DNMT3) [108]. While DNA methylation can be dynamically regulated in response to various stimuli, it is generally stably maintained in many cases, particularly in differentiated cells where it plays a crucial role in determining cell identity and fate. DNA demethylation is limited to only a few specific loci in differentiated cells, and occurs through specific mechanisms that involve the activity of TET (ten-eleven translocation) family of enzymes in conjunction with other factors [108]. However, the process of systematic TET-determined DNA demethylation is known to occur during early embryonic development, particularly after zygote formation, where it plays a critical role in erasing epigenetic marks and enabling the establishment of new gene expression patterns [108]. Additionally, DNA methylation may be passively lost through cellular replication cycles due to a basal inefficacy of the maintenance DNMT1, and this phenomenon has been utilized as a marker of biological aging of different tissues in mammals [47].

Histone modifications include post-translational chemical modifications, which occur predominantly at histone N-terminal tails and primarily involve the amino acids lysine and arginine [7, 61]. Acetylation status of histones is related to the activity of two families of enzymes, known as histone acetyltransferases (HATs) and histone deacetylases (HDACs) that add and remove one or more acetyl groups mainly at lysine residues on the histone tails [61]. Histone methylation/demethylation is catalysed by histone methyltransferase/ demethylases and can occur on both lysine and arginine residues of histone tails. In contrast to histone acetylation, which unequivocally leads to transcription activation, histone methylation can increase or decrease transcriptional activation depending on the location of the targeted amino acid residues in the histone tail and the number of methyl groups added [39].

## Epigenomics in heart failure

Complex diseases, in particular CVD, are characterized by cellular biochemical and morphological remodelling. Changes in cell identity such as dedifferentiation are permitted by wholesale reprogramming of the epigenome and the transcriptome. Targeting the key epigenetic *erasers, writers* or *readers* responsible would affect disease-associated gene regulation on a global scale, ideally reversing a number of pathophysiological pathways rather than just a single protein or pathway.

DNA demethylating agents and histone deacetylase inhibitors are already on the market and FDA-approved for the treatment of certain forms of cancer, and further are being developed [19, 37, 83, 96]. Pharmacological advances in the generation of highly specific and effective inhibitors and activators of epigenetic mediators are needed along with targeting strategies for epigenetic drugs to move closer to clinical reality.

DNA methylation has been reported as important in cardiovascular development and disease. However, despite their biological relevance, the impact of DNA methylating and de-methylating enzymes, only a few pieces of evidence accumulated that these epigenetic enzymes may play an important role in CVD [38, 80, 103, 113] Table 1]. Interestingly, recent reports outlined a new layer of regulation of TET function. It has been recently found that dysmetabolic conditions, such as diabetes, may alter DNA demethylation in association with TET relocation outside the nucleus, a phenomenon dependent on AMPK activity [124]. Whether TET extra-nuclear compartmentalization occurs in the heart of diabetic patients and whether this phenomenon has a role in this context is currently unknown.

**Table 1:** Epigenetic modification alterations in the cardiovascular system and related tissues

Modification	General function	Models	References
H3K9me2	Gene repression at promoters	Rat ascending aortic constriction	[107]
H3K4me2	Context-specific	Human dilated cardiomyopathy	[43]
		Transverse aortic constriction (TAC) in mice	[55]
H3K9me3	Gene repression at promoters	Vascular smooth muscle cell (VSMC) inflammation	[90]
		Conditional JMJD2A knockout and overexpression and TAC in mice	[63]
H3K9me2/3	Gene repression at promoters	Human failing myocardium	[134]
H3K9me3 and DNA methylation	Gene repression at promoters	Hypertrophy produced by TAC in mice and human LV hypertrophy	[46]
H3K27me3	Gene repression at promoters	Vascular smooth muscle cell (VSMC) inflammation	[43]
		Human ischemic cardiomyopathy, peri-infarct zone MI mouse hearts	[79]
H3K79me2/3	Gene silencing	Conditional DOT1L knockout in mice and human ischemic cardiomyopathy	[135]
H3S10phos	Transcriptional activation, linked to early response gene activation	CaMKIIδ knockout and TAC in mice	[40]
H3S28phos	Transcriptional activation, linked to early response gene activation	MSK1/2 knockout and isoproterenol-induced hypertrophy	[5]
DNA methylation	Gene repression at promoters. Activation in gene bodies	Human heart failure/cardiomyopathy	[33, 34, 42, 60, 69, 85, 86, 93, 95]
		Human Chagas disease cardiomyopathy	[64]
		TAC in mice	[14, 87]
		Isoproterenol-induced heart failure in mice	[16]
		Anthracycline-induced cardiac damage	[125]
		Arterial calcification in female mice	[71]
		VSMC calcification in human and rat	[70]
		Acute myocardial infarction (AMI) in mice	[75]
		Homeostasis and contractility in iPSC-derived cardiomyocytes	[77]
		Cardiomyocyte development, maturation and TAC in mice	[38]
DNA hydroxymethylation	Context-specific, gene activation	VSMC injury and human atherosclerosis	[74]

Whilst DNA methylations are important epigenetic marks, covalent modifications on histone tails work in concert to affect chromatin structure and gene expression regulating progression of CVD. Members of the HDAC Class IIa family—HDAC5 and HDAC9—protect against hypertrophic remodelling. The genetic disruption of specific genes in mice results in heightened vulnerability to cardiac hypertrophy, as well as a weakened response to stimuli that contribute to the development of hypertrophy, such as calcineurin activation and increased pressure. The binding of HDAC5 and HDAC9 leads to inhibition of Mef2c, a transcription factor that upregulates pro-hypertrophy genes [132]. When exposed to a pro-hypertrophic stimulus, two stress-responsive kinases, CaMK and PKD, act upon HDAC5 and HDAC9 causing HDAC4-dependent phosphorylation. Phosphorylated HDACs then associate with the chaperone protein 14-3-3 and relocate from the nucleus to the cytoplasm. This release of HDAC5 and HDAC9 from Mef2c leads to their interaction with p300, and consequently transcription activation [58]. This mechanism could also be relevant for the cardiotoxic effects of doxorubicin. Namely, doxorubicin impairs cardiac function by modulating the activity or expression of important Ca^2+^ handling proteins, leading to altered calcium function, and activates CaMKIIδ resulting in hyperphosphorylated PLN-T17 and RYR2-S2814, which increases the open probability of the RYR2 and leads to a significantly augmented diastolic sarcoplasmic reticulum Ca2 + leak [44]. Weather this mechanism can directly influence HDACs and to what extent that play role in the cardiotoxicity remains to be determined.

Furthermore, a complex mechanism has recently been deduced by which prevention of O-GlcNAcylation (post-translational addition of β-linked N-acetylglucosamine) to (Ser)-642 of HDAC4 stopped proteolytic cleavage of HDAC4 to create a short N-terminal fragment of HDAC4 [62]. Reducing the production of the NT HDAC4 fragment protected against cardiac hypertrophy in the diabetic heart. What’s more, preventing the production of NT-HDAC4 attenuated the pro-hypertrophic Ca2 + /calmodulin-dependent protein kinase II-mediated phosphorylation at HDAC4 Ser-632.

Thus far, less is known about the role of altered histone methylation marks in cardiac hypertrophy and disease than that of the aforementioned histone tail acetylation marks. Thienpont et al., identified a mechanism by which binding of miR-217 to a non-canonical binding site in the untranslated region of euchromatic histone lysine methyltransferases (EHMTs) in CMs in response to pathological stimuli caused a reduction in H3K9me2 in hypertrophy-associated genes such as *Nppa, Nppb* and *Myh7* [107]*.* Preventing the miR-217-induced loss of EHMT and H3K9me2 genetically or using antisense oligo therapy attenuated the cardiac hypertrophy in vitro or in vivo models.

Assembly of the repressive chromatin complex BRG1–G9a/GLP–DNMT3 at the adult myosin heavy chain (Myh6) gene promoter, with increased H3K9 and CpG methylation at the locus, has been associated with gene repression in pressure overloaded stressed mouse hearts [43]. Chromatin structure is determined by nucleosome positioning, histone modifications, and DNA methylation. How chromatin modifications are altered under pathological conditions (in a coordinated manner) remains elusive. In human hypertrophic hearts, the BRG1-G9a/GLP-DNMT3 complex is also activated; its level correlates with H3K9/CpG methylation, Myh6 repression, and cardiomyopathy.

## RNA editing and the epitranscriptome in heart failure

RNA editing represents a set of different enzymatically mediated modifications of ribonucleotides within coding and non-coding RNA molecules. Some of the most prevalent types of modifications includes methylation of different nucleotides and conversion of one nucleotide to another. Although the first modified nucleoside—5-ribosyluracil, or pseudouridine—was discovered in the 1950s, studies have only recently been aimed at understanding the role of RNA modifications in the epigenetic control of pathophysiological responses in the cardiovascular system. Indeed, the chemical modification of mRNA, tRNA, and rRNA was once deemed to be a static phenomenon involved largely in fine-tuning the structure of those macromolecules. However, ribonucleic acid bases in coding and noncoding RNA can be modified in a dynamic, reversible manner that is suggestive of a regulatory code atop the primary sequence. In analogy with epigenetics, the effector proteins that lead to epitranscriptomic modifications are also referred to as *writers*, *erasers* and *readers* [129]. The *writers* introduce certain types of modification in RNA, while the *erasers* recognize these modifications and catalyse their removal. However, the downstream effects of these structural changes depend on the *readers* [129], the proteins that specifically interact with modified bases thus affecting RNA behaviour and resulting in defined downstream effects [129].

Epigenetic modifications of RNA are modifications to the structure of the RNA molecule that include both methylation and hydroxymethylation of the cytosine base, as well as modifications of the adenine base. As for DNA, RNA methylation is the addition of a methyl group to the cytosine base, which changes the structure of the RNA molecule and can have an effect on gene expression [53]. Hydroxymethylation of the cytosine base is similar to methylation, but the hydroxymethyl group is added instead of the methyl group. This modification is especially abundant in cardiac tissue [29, 31]. Modification of the adenine base can also occur and can be a form of epigenetic modification. This involves the addition of a methyl or hydroxymethyl group to the adenine base, which can also affect gene expression [24, 82, 117, 118]. Epigenetic modifications of RNA are important for gene expression and have been studied extensively in recent years [73].

Specifically, there are a number of RNA epigenetic modifications of which the most common include N6-methyladenosine (m6A), N5-methylcytosine (m5C), N1-methyl Adenine (m1A), N6,2′-O-dimethyladenosine (m6Am), N7-methyl guanosine (m7G) and the Cytosine hydroxylation [22, 121].

**Fig. 2:**
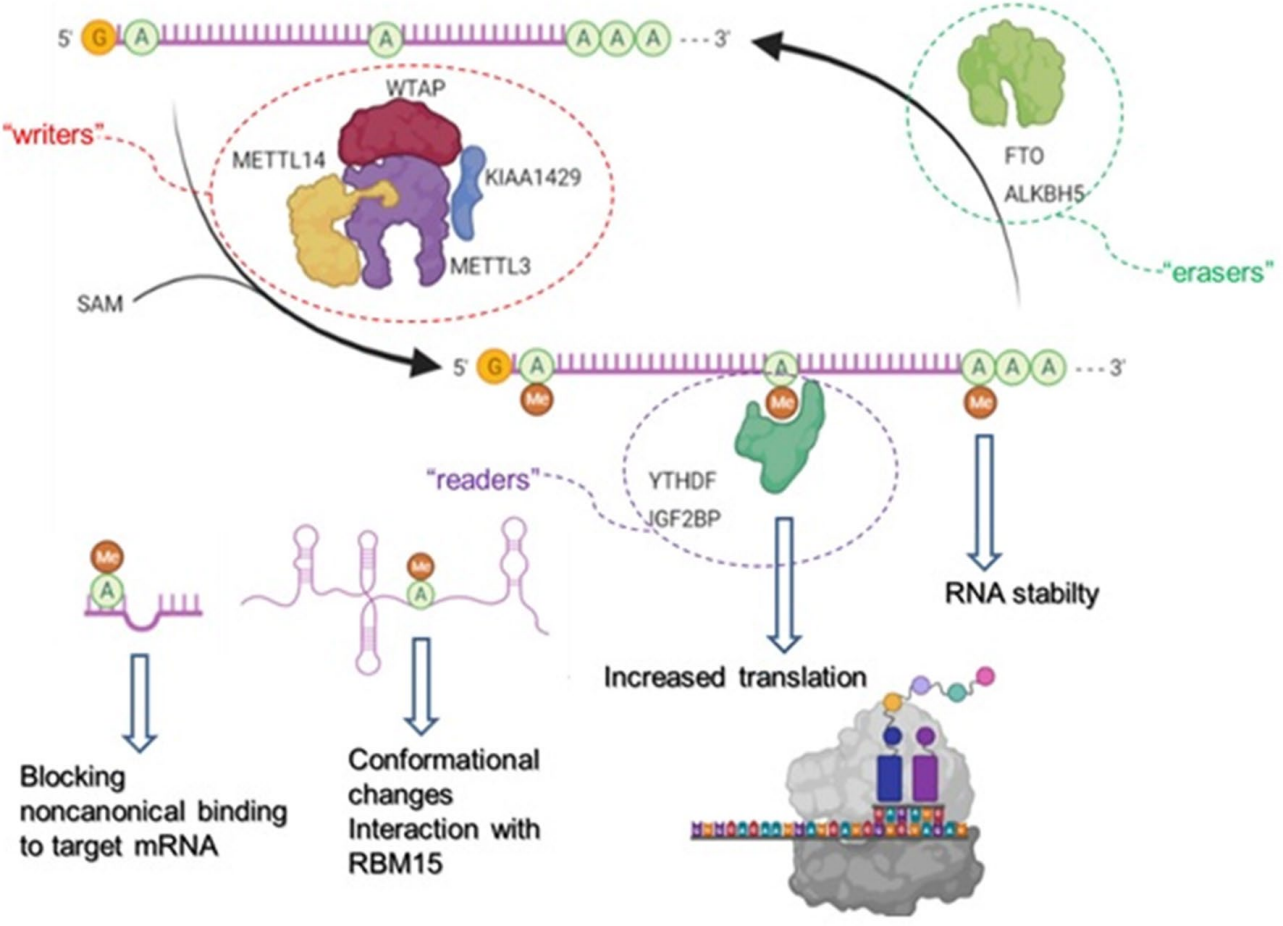
The most important players in m6A RNA modifications and possible downstream effects. writers, erasers and readers (created by Biorender). *METTL3* methyltransferase 3, *METTL14* methyltransferase 14, *WATAP* Wilms tumour‑associated protein‑1, *FTO* fat mass and obesity-associated protein, *ALKBH5* α-ketoglutarate-dependent dioxygenase alkB homolog 5, *YTHDF2* YT521-B homology domain, *IGF2BP* insulin-like growth factor-2 mRNA-binding protein

For the scope of this article we will focus on m6A that is considered to be the most prevalent epitranscriptomic modification, found on ~ 25% of transcripts at the genome-wide level (Fig. 2). It is most frequently located near the stop codons, the 5´ and 3´-UTR, and within long internal exons affecting the RNA stability, translation, and secondary structure formation [23]. M6A is produced in the reaction catalysed by the multi-subunit *writer* complex that adds a methyl group to the N6 position of adenosine [129]. This complex includes methyltransferase 3 (METTL3), which plays a catalytic role, methyltransferase 14 (METTL14) that acts as a RNA-binding domain, and the cofactors Wilms tumour‑associated protein‑1 (WATAP) and KIAA1429 [114, 116]. As a reversible modification, m6A can be demethylated by *erasers* such as fat mass and obesity-associated protein (FTO) and α-ketoglutarate-dependent dioxygenase alkB homolog 5 (ALKBH5) [54, 89]. The biological effects of m6A modifications go beyond their influence on RNA structure and stability. As mentioned above, these effects are mediated through the interaction with *readers* such as the YT521-B homology domain (YTHDF2) and the insulin-like growth factor-2 mRNA-binding protein (IGF2BP) that recognize and bind to specific m6A modifications [48, 138].

So far, several studies have demonstrated a critical role for m6A modifications in heart function. Namely, cardiac hypertrophy is associated with a significant increase in m6A methylation of mRNA isolated from cardiomyocytes (CMs), with enrichment in specific functional clusters of genes, such as those regulating kinases and intracellular signalling pathways [26]. In addition, through experiments taking advantage of METTL3 overexpression, it appears that METTL3 drives compensated hypertrophy in vivo. Moreover, following ischemia/reperfusion, upregulation of METTL3 has been implicated in the control of genes related to autophagy [91, 98, 99]. M6A hypermethylation of mRNA encoding for myosin regulatory light chain 2 is observed in failing hearts, and leads to a decrease in protein levels [59]. Coherently, inhibition of METTL3 blunts CM hypertrophic growth whereas increased expression of METTL3 is sufficient to stimulate its development [25]. In the intact animal, METTL3 overexpression induces adaptive growth of the heart without untoward functional effects whereas cardiac-specific knockout leads to heart failure upon stress or aging. These findings are indicative of a role for RNA methylation in maintaining cardiac homeostasis [33]. Additionally, FTO is downregulated in the failing heart, a phenomenon associated with increased deposition of m6A on CM mRNA and worsened contractile function [81, 94]. Indeed, FTO selectively demethylates transcripts encoding proteins involved in muscle contraction, sarcomere organization, and cardiac hypertrophy, preventing their degradation. On the other hand, overexpression of FTO is associated with reduced cardiac fibrosis and increased angiogenesis in mice with myocardial infarction, suggestive of a possible therapeutic value in targeting this mechanism [81].

M6A modification can also influence the functions of ncRNAs. For example, during miRNA biogenesis, pri-miRNAs are modified by METTL3-depending mechanism [2, 3]. These m6A modifications are recognized by “reader” heterogeneous nuclear ribonucleoproteins A2/B1, which in turn, stimulate the initiation of DICER-mediated processing through the recruitment of DGCR8 [111]. It seems that m6A modifications block A:G non-canonical base-paring, affecting the strength of miRNAs interaction with target mRNA [38]. In addition, two lncRNAs associated with MI, X‑inactive specific transcript (XIST) and Metastasis associated lung adenocarcinoma transcript 1 (MALAT1) are highly prone to m6A modification. XIST-mediated transcriptional repression of X-linked genes is dependent on m6A modification and binding of m6A “reader” RNA binding motif protein 15 (RBM15) [92]. In the case of MALAT1, m6A modifications induce conformational changes and increased binding by a number of RNA binding proteins (RBPs) [101]. Adenosine deaminase acting on RNA (ADAR1) also seems to play important role in heart function and development. In neonatal mice CMs, oxidative stress induced simultaneously ADAR1 and protein kinase PKR expression leading to increased apoptosis and inflammation [120]. However, overexpression of ADAR1 inhibited PKR activation, which is suggested to be a self-preservation mechanism in neonatal CMs that limits excessive apoptosis and inflammation. In the study by Moore et al., cardiac knockout of ADAR1 is associated with embryonic lethality leading to the conclusion that ADAR1 is essential for embryonic CM survival and proliferation [84]. In the same lines, Azzouzi et al. have induced an ADAR1 deletion in adult CMs through the use of a tamoxifen-inducible Cre recombinase under the control of the cardiac-specific α-myosin heavy chain promoter (αMHC) [27]. The study shows that targeted ADAR1 deletion leads to a significant increase in ADAR1‑null mice lethality, accompanied by induction of stress markers, overall reduced expression of miRNAs, severe ventricular remodelling and quick and spontaneous cardiac dysfunction, mediated through miR-199a-5p and unfolded protein response. In addition to coding RNAs, A-to-I editing plays important role in the function of miRNAs. So far, 2711 potential pri-miR editing sites have been described within approximately 80% of all human pri-miRs, implicating that A-to-I editing can have a tremendous impact on miRNA target specificity [67]. Increased A-to-I-editing of vasoactive miR-487b-3p has been found in ischemic muscle tissues undergoing neovascularization after induction of hind limb ischemia [110]. The edited mature miR-487b-3p has a unique targetome and promotes angiogenesis, in contrast to the canonical miR-487b-3p. It seems that vasoactive microRNA editing is a widespread phenomenon that enhances neovascularization in response to ischemia [119]. Other RNA modifications such as m1A, m3C, m5C, m7G, and their roles in the pathophysiology of hear diseases have been highly understudied.

## Epigenetic and epitranscriptomic crosstalk

Interactions between the “epi” worlds of DNA and RNA have not been extensively studied. However, lately, new evidence is starting to reveal the importance of the interplay between epigenetic and epitranscriptomic mechanisms in the regulation of gene expression.

M6A writing complex can influence histone modification status and chromatin state indirectly by inducing m6A modifications to mRNAs transcribed from the genes that encode for epigenetic modifiers, affecting their stability and expression levels [11]. M6A hypomethylation of EZH2 and CBP/p300 mRNA through METTL3 and METTL14 knock-out in neural stem cells leads to a decrease of H3K27me3 (marker of transcriptional repression) and an increase of H3K27ac (marker of transcriptional activation) compromising the ability of the cells to proliferate [17, 119]. In mouse embryonic stem cells, METTL3 silencing leads to the elevation of two histone marks associated with active transcription (H3K4me3 and H3K27ac), resulting in increased chromatin accessibility and chromatin-associated regulatory RNAs (carRNAs), including promoter-associated RNAs, enhancer RNAs, and repeat RNAs [72]. Furthermore, Li et al. have studied how epigenetic signals respond to m6A methylation in human Flp-In HEK293 cells [68]. Knockdown of METTL3 results in a significant increase in H3K9me2 and minor upregulation of H3K27ac and H3K27me3 [49]. Besides indirect effects on epigenetic control, m6A writing complex can directly bind to chromatin remodelling factors and histone modifications. Direct binding of METTL14 to H3K36me3 brings the whole m6A writing machinery in near proximity of RNA polymerase II thus enabling the addition of m6A to nascent RNAs during transcription [115]. On the other hand, epigenetic mechanisms can also play important roles in the regulation of epitranscriptomic mechanisms. Histone acetyltransferase p300 increased H3K27 acetylation of METTL3 promoter, leading to increased METTL3 transcription and m6A hypermethylation [133]. Furthermore, decreased methylation of METTL3 promoter results in METTL3 downregulation and reduced m6A levels on pri-miR-25, disrupting its maturation [18]. Similarly, the demethylation of H3K4me3 in the METTL14 promoter by the lysine-specific histone demethylase 5C promoted the decreasing the transcription of METTL14 [127].

M6A readers are also able to take an active part in epigenetic-epitranscriptomic interplays. In the nucleus, m6A-marked intracisternal A particle (IAP) transcripts interact with YTHDC1, which recruits METTL3 and in turn promotes the association of METTL3 with chromatin [105]. In addition, METTL3 interacts with histone 3 lysine 9 (H3K9) tri-methyltransferase SETDB1 and its cofactor TRIM28, important for their localisation to IAPs [105]. YTHDC1 binds m6A-modified carRNAs leading to their degradation and decreased chromatin accessibility [72]. YTHDC1 promotes the demethylation of H3K9me2 through interaction with lysine demethylase 3B (KDM3B), ultimately leading to increased gene expression [68]. In the cytoplasm, another m6A reader, YTHDF2, destabilizes m6A methylated lysine demethylase 6B (KDM6B) mRNA leading to an increase in H3K27me3 levels [123]. On the other hand, miRNa-145 binds to the 3’-UTR of the YTHDF2 mRNA, resulting in its downregulation. Another interesting level of interplay is observed in smooth muscle cells under high-glucose conditions. Namely, circYTHDC2 targets the 3’-UTR of the TET2 mRNA leading to its degradation and in turn influence methylation of the DNA [128].

Lastly, m6A erasers that decrease m6A levels in RNAs can ultimately influence histone marks via several mechanisms. ALKBH5 decreases m6A levels on lncRNA TRERNA1, then in turn act as a scaffold and recruit EZH2 leading to the increase in H3K27me3 modification of cyclin-dependent kinases inhibitor p21 promoter and the decrease of its expression [100]. In addition, ALKBH5 knockdown leads to the increase of H3K9me3 levels in mouse embryonic stem cells via unknown mechanisms [127].

**Fig. 3:**
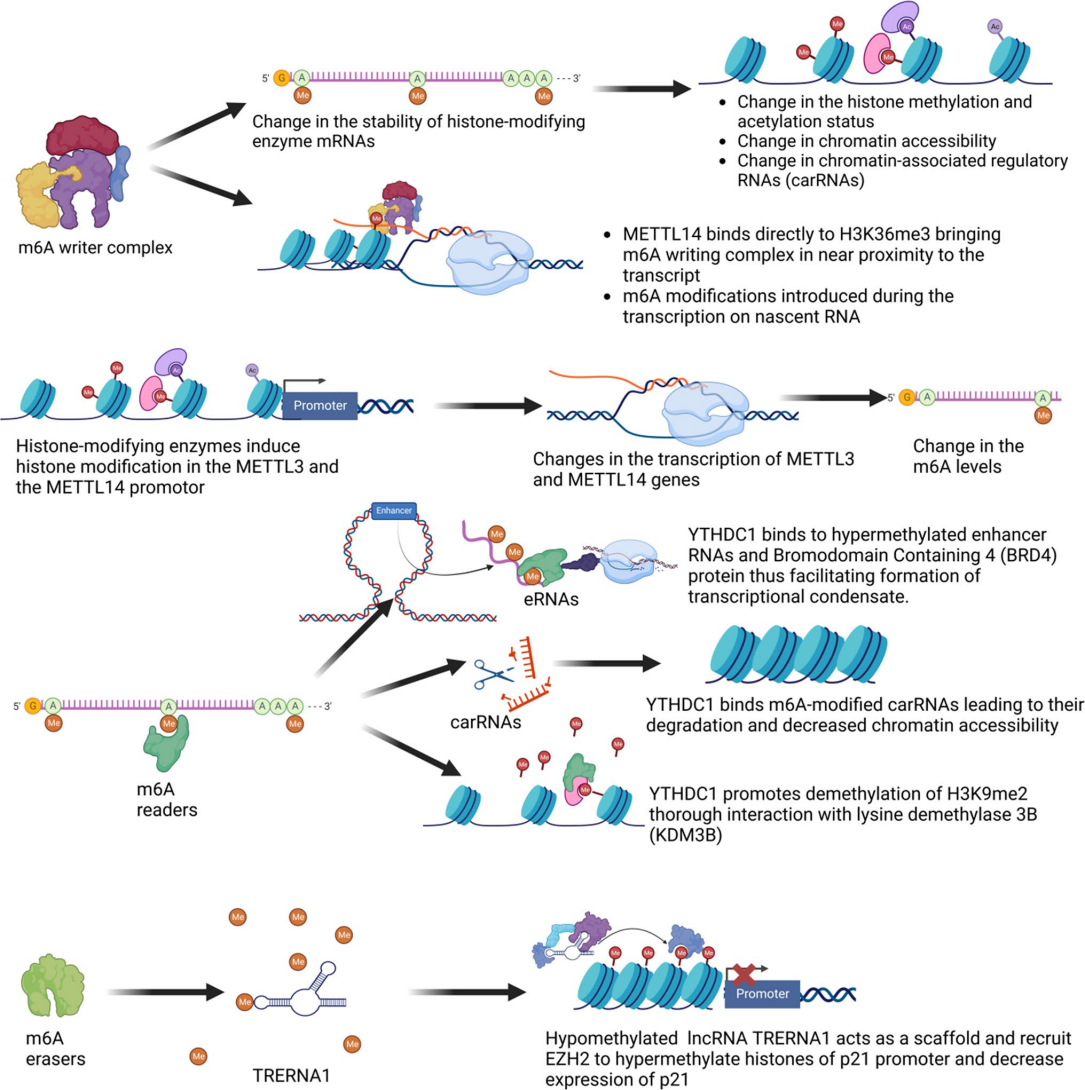
Mechanisms of epigenetics-epitranscriptomics cross-talk (created by Biorender). *METTL3* methyltransferase 3, *METTL14* methyltransferase 14, *YTHDC1* YTH domain-containing protein 1, *EZH2* enhancer of zeste homolog 2

The presented data strongly suggest that the cross-talk between epigenetic and epitranscriptomic mechanisms represents an important level of cellular regulation that should be more extensively explored, particularly in the context of heart disease. So far, we do not have clear information how these mechanisms could be important in the function of the different cardiac cell types. Thus, it seems important to drive the field in this direction and reveal how these complex interplays can contribute to our better understanding of the pathological process behind cardiac dysfunction. Mechanisms of epigenetics-epitranscriptomics cross-talk are summarized in Fig. 3.

## Integration of epigenomics data through machine learning algorithms

To untangle the complex biology of cellular mechanisms, and differences in multiple cells, tissues and organs caused by hear failure, multi-omics measurements are often necessary. Multi-omics analyses adopt a holistic view toward the universal detection of genes (genomics), epigenetic modification (epigenomics), RNA expression (transcriptomics), protein translation (proteomics) and metabolite production (metabolomics) that can characterize eventually specific sets of biological samples in a non‐targeted and non‐biased manner. The rationale of these approaches is that a complex system can be understood more thoroughly if considered as a whole. By integrating epigenomics with other regulatory layers, we integrate data in a broader context that improves our understanding of the complex pathophysiological responses at the origin and progression of cardiac dysfunction.

There are many computational approaches for the analysis of multi-omics data, reviewed in [4, 10, 57]. Several integration strategies have been proposed to ensure that the main methodological challenge of multi-omics integration is addressed, namely ensuring that the information contained in each molecular “view” is used in a complementary rather than conflicting way, and does not overshadow the information present in other omics. Network-based, model-based and machine-learning based approaches have been proposed to solve this issue, with successful results [45, 66, 109]. As with experimental data, the accessibility of models to the community is of paramount importance. The model developers should be encouraged to use established frameworks such as the Systems Biology Markup Language (SBML) to facilitate dissemination [50].

While the use of machine learning methods has generated much interest, it is often limited by a lack of appropriate training data. Furthermore, several other challenges remain, e.g. the presence of redundant information across the omics, and an effective strategy for interpreting the machine learning models while integrating the omics data. Despite these challenges, using bioinformatics and systems biology tools, researchers can make informed decisions, and develop models that automatically learn and improve from feedback. In this machine learning framework, both “supervised” and “unsupervised” methods can be used. In supervised learning, one aims to predict target functions associated with a sample (e.g. risk score associated with a patient). This can be achieved through classifiers, when the goal is to predict the class to which a new sample belongs (e.g. high-risk or low-risk patients), or regressors, when the goal is to predict a numerical quantity (e.g. an exact risk score for each patient). Conversely, in unsupervised learning, one aims at predicting latent associations or patterns among the variables. In this context, clustering techniques can reveal patterns in the data, while dimensionality reduction techniques (e.g. principal component analysis, multi-dimensional scaling) can elucidate the key contribution to the variation observed in CVD data and to the interactions with other diseases like type 2 diabetes [8]. Merging biological knowledge with in silico models has been shown to contribute towards biologically informed and interpretable machine learning methods, even with regularised regression techniques [78].

Supervised machine learning is a powerful technique when the response “labels” are available for each of the samples used to improve the model. With such labels, a model can learn both from data and from feedback on the predictions it makes. One of the first examples of machine learning in the context of CVD was a semi-supervised class discovery method that was developed to predict clinical parameters and risk factors [28]. Recently, data from a large cohort of 378,256 patients was fed to four machine-learning algorithms to predict the first cardiovascular event over 10 years, with the best performance achieved by neural networks [122]. Machine and deep learning tools can be also used to discover patterns from large and heterogeneous data like those obtained in epigenomics studies, thus deepening and enhancing our understanding of interplay between different layers of epigenomic landscape. Using deep learning, Zhao et al. developed a model for the prediction of heart failure with preserved ejection fraction that was based on the methylation profile of 25 DNA loci and 5 clinical features (age, diuretics use, body mass index, albuminuria, and serum creatinine) [136]. The developed method outperformed models including only clinical features or DNA methylation data. In another study, the use of machine learning approaches enabled the prenatal detection of foetal congenital heart defects based on methylation profiles in circulating cell-free DNA from maternal blood [6]. In the study by Fernández-Pérez et al., different machine learning models (elastic net regression, K-Nearest neighbours, random forest, and support vector machine models), and one deep learning approximation (multilayer perceptron model) were used to explore the influence of different clinical and lifestyle factors on global DNA methylation profiles in patients with cerebrovascular disease [30]. The best models were elastic net regression and multilayer perceptron model, although with modest capability, explaining around 35–40% of variability in methylation profiles. By using cross-combination of three machine learning methods, including best subset regression, regularization techniques, and random forest algorithm, Ma et al. discovered a signature of five genes that acts as m7G regulators that can distinguish patients with heart failure from healthy subjects [76]. As previously mentioned, added value of machine learning approaches is their ability to ingrate data that come from different levels of cellular complexity, so-called multi-omics data. Zhou et al. used a multi-omics approach to explore molecular signatures in a mouse model of heart failure [137]. This study included machine learning-based integrative analysis of scRNA-seq, scATAC-seq, bulk ATAC-seq and miRNA-seq data that provided mechanists insights in the complexity of pressure overload-induced heart failure.

When applying bioinformatics or systems biology tools, the quality of pre-processing and data control is the key to avoiding learning and propagating noise in the machine learning model. Effective feature engineering or feature selection, and attempting different strategies for data integration, are also paramount to extracting cross-omics information from the different omics variables. Different omics layers may require different omics-specific data extraction strategies. After extracting latent information from each omics, a final integration method is often required to collate the latent multi-omics information, discarding any partial overlap (e.g. between genomics, epigenomics, transcriptomics, proteomics and metabolomics). This multi-step approach (also called transformation-based or intermediate-stage omics data integration) has often proven successful when dealing with multi-omics data [21], but further research is needed to ensure that the information overlap between the omics can be reduced, and only the unique information that each contributing omics can be extracted before combining all parameters.

**Fig. 4:**
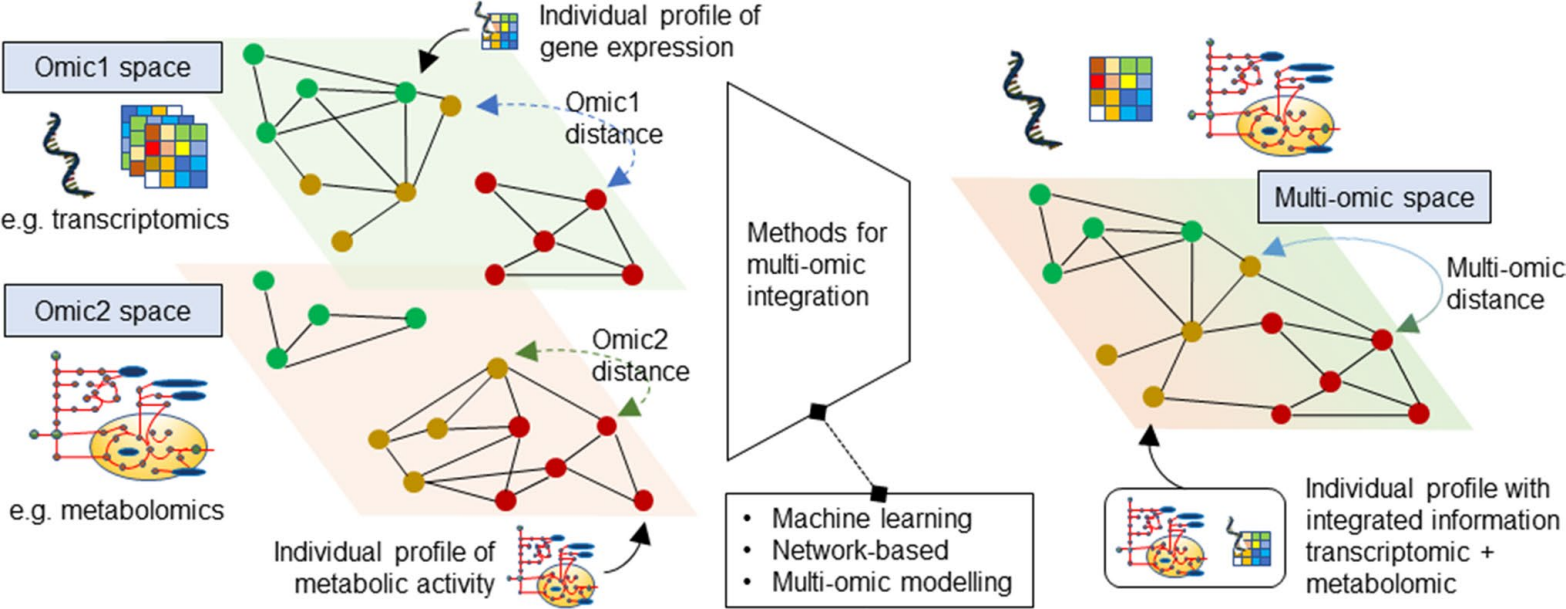
A single profile (e.g. a patient) can be measured on different omic spaces (e.g. transcriptomic data and metabolic activity). The distance between two patients can therefore also be measured in different spaces. The distance according to the transcriptomic profile of the two patients is likely to differ from the distance between the same two patients, but measured according to metabolism. Methods for integration of multiple omics define a multi-omic distance, e.g. by combining the distances from the individual omic profiles, therefore providing a systems-level view of the omic profiles. This strategy mitigates the bias that a single-omic analysis may introduce

To better exploit predictive algorithms and avoid using them as “black boxes”, incorporating mechanistic information seems a promising direction for the research field. In this regard, information derived from cell modelling can be incorporated into machine learning pipelines, for instance at the stage of feature selection. The availability of genome sequences, combined with biochemical information, has enabled the reconstruction of genome-scale models of metabolism. These include all known metabolic reactions in the cell, and can also have different coverage and scope, e.g. integrating metabolism with regulation, signalling, protein interactions and further cellular routes [88]. For instance, CardioNet was the first reconstruction of the metabolic network of the human cardiomyocyte [56]. Deep learning, and in particular its multi-modal versions, have been proposed to learn from these combined multi-omic datasets, including those generated by metabolic models [130]. Simulation of these metabolic models can be incorporated into the machine learning methods, therefore providing biomarkers and useful information that make the machine learning predictions more interpretable (see Fig. 4).

## Conclusions and perspectives

Major and rapid advances in next-generation sequencing technologies to study the transcriptome and epigenome have greatly enhanced our understanding of the molecular basis of disease in cells and tissues of the cardiovascular system. A large number of groups have now published bulk or single-cell sequencing data from human heart samples and pre-clinical models of cardiovascular disease. The number of publications in PubMed describing “cardiovascular disease,” “RNA-sequencing,” and “epigenomics” has increased from a total of 21 to 322 in the last decade. These data sets have undoubtedly expanded our knowledge and understanding of disease mechanisms in cardiovascular disease and leads to promising trials of epigenetic drugs (e.g. NCT05350969) [9, 103, 104].

One major challenge associated to the widespread generation of big datasets is the unification of methodologies used to analyse data to be able to compare and integrate results generated by different research teams. This requires formulation of standardization of tools and analytical and appropriate education and training of individual researchers as well as minimal requirements for methodological reporting and data storage. Currently, none of these measures are in place, leading to poor reproducibility of data and massive underutilization of publicly available data, often generated from precious human specimens. The lack of consensus in sample storage, next-generation sequencing library preparation, data storage and analysis as well as thorough reporting of all the aforementioned is being recognized as holding back clinically important and relevant findings in the cardiovascular community [94].

Another aspect to consider is that alterations in chromatin, DNA modifications and RNA expression may be more consequences of disease rather than being the actual promoting factors for disease initiation and progression. Epigenetic and transcriptomic changes can be therefore adaptive in response to the pathological stressors. These mechanisms could be harnessed to protect the heart and vasculature in a disease context or even to prevent the detrimental effects of exposure to risk factors. Nevertheless, some modifications are disease-driving. Big data, including RNA-sequencing and epigenomics data, can provide an in-depth analysis of the state of cells or tissues at a given point in time or stage of disease progression.

A novel useful approach in studying epigenetic modifications can be through the use of gene editing tools based on CRISPR/Cas9 system. Modified Cas9 named dCas9 can be fused with genetic modifiers and used to introduce locus-specific modifications such as DNA methylation, histone methylation and acetylation [13]. Another application of this technology is live cell chromatin imaging, which allows to visualize specific locations in the genome in real time. This can be achieved by labelling dCas9 with fluorescent tags, which enables them to see the genomic loci in a single-, dual-, or multicolor way [13]. In addition, the use of Cas13 enzymes that bind to RNA and not DNA, opens new doors for site-specific epitranscriptomic research [13].

In order to distinguish between these various forms of molecular alteration and to identify relevant targets to guide drug development, it is essential to combine big data acquisition with functional cellular and pre-clinical studies using appropriate models. This validation requires a multi-disciplinary approach to translational cardiovascular disease, bringing together clinical and basic scientists, bioinformaticians and data scientists with molecular biologists, biochemists and medicinal chemists and experience animal technicians. These specialities are seldom found within a single research group. In this way, working in an interdisciplinary, collaborative manner, bringing together different expertise, such as that provided by the EU-CardioRNA COST Action, optimizes the opportunity to make breakthroughs with more sensitive and disease-specific diagnostic tools and treatments.
